# Technical note: subscapularis-sparing approach to perform anatomic total shoulder arthroplasty using a multiplanar humeral osteotomy and angled glenoid instruments

**DOI:** 10.1186/s13018-021-02900-w

**Published:** 2022-01-11

**Authors:** Sohil S. Desai, Ryan A. Nelson, Kayla C. Korbel, William N. Levine, Steven S. Goldberg

**Affiliations:** 1 Department of Orthopedic Surgery, Columbia University Medical Center, New York, NY USA; 2 Physicians Regional Medical Center, Naples, FL USA

**Keywords:** Total shoulder arthroplasty, Subscapularis-sparing, Stemless shoulder, Glenohumeral osteoarthritis

## Abstract

**Background:**

Anatomic total shoulder arthroplasty is typically performed through the deltopectoral approach followed by either a subscapularis tenotomy, tendon peel, or lesser tuberosity osteotomy to provide adequate exposure. These subscapularis-takedown methods have been associated with incomplete subscapularis healing, however, and as a result often lead to functional deficits and complications. Subscapularis-sparing approaches have been introduced to mitigate these complications, but thus far have either been limited to hemiarthroplasty or resulted in residual inferior humeral head osteophytes and humeral component size mismatch. The present technique demonstrates the possibility for surgeons to capitalize on the improved patient outcomes that are afforded by subscapularis-sparing approaches, while still utilizing the deltopectoral interval to perform a total glenohumeral joint arthroplasty.

**Methods:**

This article describes in detail the placement of a stemless anatomic TSA with the use of angled glenoid instruments through a subscapularis-sparing deltopectoral approach. Postoperatively, patients are placed in a sling but are instructed to remove as tolerated, as early as the 1st postoperative week. Physical therapy is started at week 1 with a 4-phase progression.

**Conclusions:**

This technique using a TSA system with a polyaxial glenoid reamer and angled pegs on the backside of the glenoid allows the potential for maintenance of the strong postoperative radiographic and patient-reported outcomes that are achieved using traditional TSA approaches, with the advantage of accelerated rehabilitation protocols and decreased risk of subscapularis insufficiency that result from the use of subscapularis-sparing approaches.

## Background

Anatomic total shoulder arthroplasty (TSA) is typically performed through the deltopectoral approach followed by either a subscapularis tenotomy, tendon peel, or lesser tuberosity osteotomy to provide adequate exposure. Each technique requires appropriate repair as well as specific rehabilitation guidelines to protect the subscapularis postoperatively. Several studies have demonstrated postoperative functional deficits and complications associated with incomplete subscapularis healing following these techniques [1–7]. Subscapularis-sparing approaches provide a potential solution to this problem of postoperative subscapularis insufficiency, but additional challenges have arisen in the form of achieving adequate access to the humerus and glenoid for bone preparation. Three subscapularis-sparing techniques have been defined by Lafosse et al., Simovitch et al., and Savoie et al., respectively [8–10].

Lafosse et al. utilize a superolateral incision, deltoid split, and dissection through the rotator interval [8]. The superior subscapularis and other rotator interval landmarks are identified, followed by a 4-cm incision along the junction of the subscapularis and anterior border of the coracohumeral ligament (CHL). A 2-cm lateral incision within the rotator interval detaches humeral insertions of the CHL and superior glenohumeral ligament (SGHL), and a 3-cm medial incision releases the CHL and SGHL from their glenoid attachments. At this point, the arthroplasty can be performed entirely through the rotator interval. Biceps tenodesis, acromioplasty, and humeral anatomic neck osteotomy are also performed. Care is taken not to violate the subscapularis and supraspinatus tendons during any portion of the exposure and implant placement [8].

Simovitch et al. utilize an incision 1 cm lateral to the standard deltopectoral approach, and achieve exposure of the glenohumeral joint through the rotator interval [9]. First, exposure of the humerus begins inferiorly, with visualization of the inferior capsule and inferior humeral neck achieved through a subscapularis window. The inferior muscular portion of the subscapularis is released from the humeral neck and retracted inferiorly, while the tendinous insertion is left intact. A biceps tenodesis is performed. At this point, the landmarks of the rotator interval are identified, and the rotator interval capsular tissue is excised from lateral to medial. After adequate soft tissue retraction, humeral and glenoid preparation can be performed through this rotator interval window. The inferior subscapularis muscle flap is left unrepaired.

Savoie et al. utilize a standard deltopectoral approach and identify the subscapularis muscle after initial dissection. A horizontal split is made in the inferior subscapularis muscle tendon raphe, roughly 1/2 to 2/3 inferior to the superior subscapularis border. A vertical incision is made from the lateral margin of the subscapularis split, releasing the subscapularis tendon from its humeral insertion. With the assistance of retractors, the humeral head is easily visualized with external rotation and abduction. Inferior humeral head osteophytes are visualized and removed, and humeral head replacement is performed. Biceps tenodesis is done in select cases, and the inferior subscapularis flap is then repaired to the superior portion as well as to the lesser tuberosity by use of a suture anchor [10].

As mentioned previously, these subscapularis-sparing approaches provide the advantage of decreased subscapularis disruption, but were limited to hemiarthroplasty in Savoie’s initial cohort, and resulted in residual inferior humeral head osteophytes and humeral component size mismatch in both the Lafosse et al. and Simovitch et al. cohorts [8–10].

Thus, in order to attain the desired decreased risk of subscapularis insufficiency, while achieving consistent and accurate postoperative radiographic outcomes, the current technique was developed. Herein, we describe in detail the technique of placing a stemless multiplanar humeral osteotomy anatomic TSA with the use of a glenoid implant and instruments specifically designed for insertion with reduced glenohumeral distraction through the Savoie subscapularis-sparing approach.

## Methods

### TSA implants and instruments

The devices utilized during this technique were a CoCr ellipsoid humerus and all-polyethylene glenoid from the Catalyst CSR Total Shoulder System (Catalyst OrthoScience, Naples, FL, USA), which received FDA approval for use in the USA in 2016. The ellipsoid humeral head is a 1-piece CoCr humerus component with a radius of curvature in the anterior–posterior axis that is 93% of the radius of curvature in the superior–inferior axis, based on anatomic studies of the humeral articular surface, most notably by Iannotti and other authors [11–15]. The glenoid component is a 1-piece all-polyethylene implant with angled pegs that is inserted at 17 degrees anterior to the glenoid articular surface. In contrast to traditional keeled and straight-pegged glenoid implants, which are inserted in a perpendicular fashion to the normal glenoid articular surface, the novel 17-degree angled design allows for glenoid preparation and implant insertion with less posterior retraction of the humerus. All retractors utilized in this technique are widely available and manufactured by Innomed, Inc. (Savannah, Georgia). No custom or specially modified retractors were necessary.

### Deltopectoral approach and biceps tenodesis

**Fig. 1:**
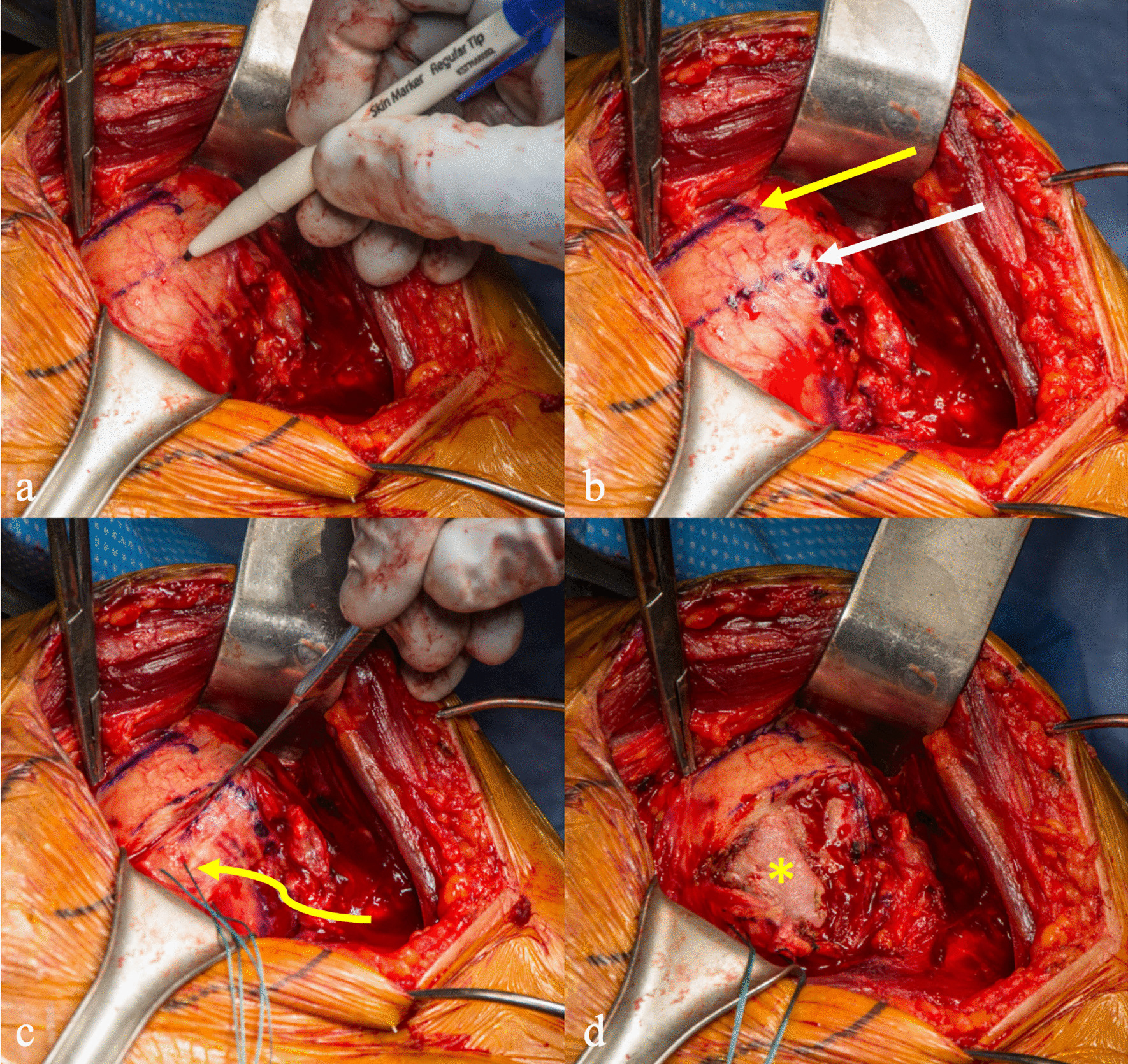
Following a standard deltopectoral dissection, **a** a Richardson retractor is placed medially and a deltoid retractor is placed under the deltoid. **b** The superior subscapularis border (yellow arrow) and planned incision of the inferior subscapularis (white arrow) are marked. **c** Incision is made along the dotted line with 2.0 Ethibond sutures (curved arrow) placed in the inferior subscapularis flap, allowing **d** inferior retraction and exposure of the humerus (asterisk)

Patients are positioned in the 45-degree beach chair position and placed under general anesthesia in combination with an interscalene block. A standard deltopectoral incision is made, and dissection is made through the interval between the pectoralis major medially and the deltoid laterally. A Richardson retractor is placed medially, and a deltoid retractor is placed under the deltoid (Fig. 1a). The cephalic vein is typically retracted laterally. The long head of the biceps can be located at the top of the pectoralis major and is released. Biceps tenodesis is performed with attachment of the biceps tendon to the pectoralis major.

### Inferior subscapularis tendon reflection

The superior border of the subscapularis is marked with a straight line, and a horizontal dotted line is marked across the subscapularis midpoint (Fig. 1a). Along the subscapularis insertion on the humerus, the dotted line is taken inferiorly just medial to the border of bicipital groove (Fig. 1b). A horizontal incision is made along the dotted line splitting the subscapularis parallel to its fibers (Fig. 1c). Next, a vertical incision along the inferior continuation of the dotted line is made to release the inferior 1/2 to 1/3 of the subscapularis from its humeral insertion. The inferior subscapularis flap is tagged with a 2.0 Ethibond suture and is reflected inferiorly exposing the articular border of the humeral head and dissection continues to release capsule off the inferomedial neck of the humerus (Fig. 1d).

### Humeral head preparation

**Fig. 2:**
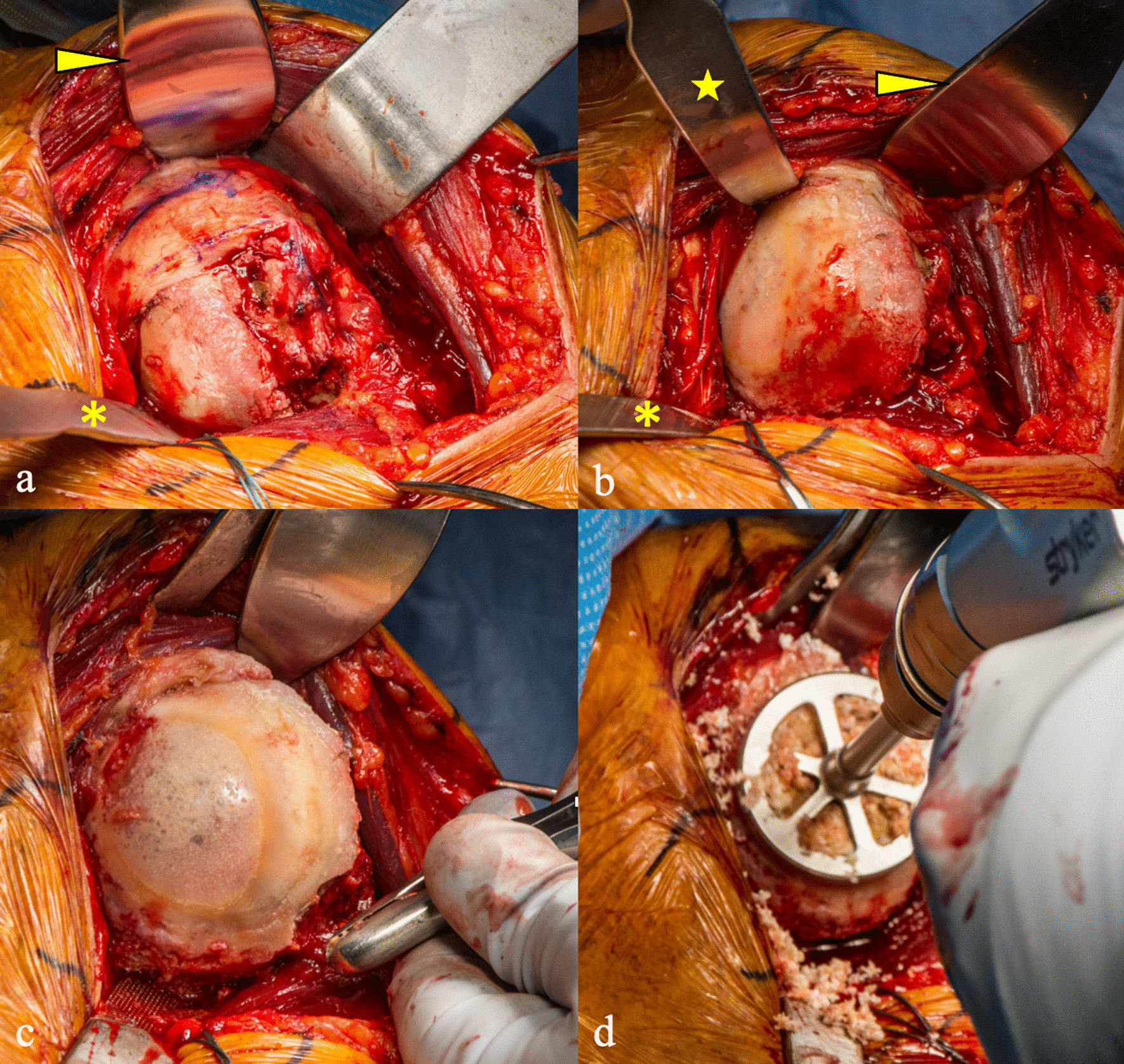
Humeral preparation begins with **a** Darrach retractor (asterisk) placed medially in the glenohumeral joint and cobra retractor (arrowhead) deep to the deltoid. **b** Chandler retractor (star) is then placed deep to subscapularis, while the arm is externally rotated. **c** Continued external rotation improves humeral head visualization, allowing osteophyte removal with a rongeur followed by **d** reaming of humeral subchondral bone over a central guide pin

**Fig. 3:**
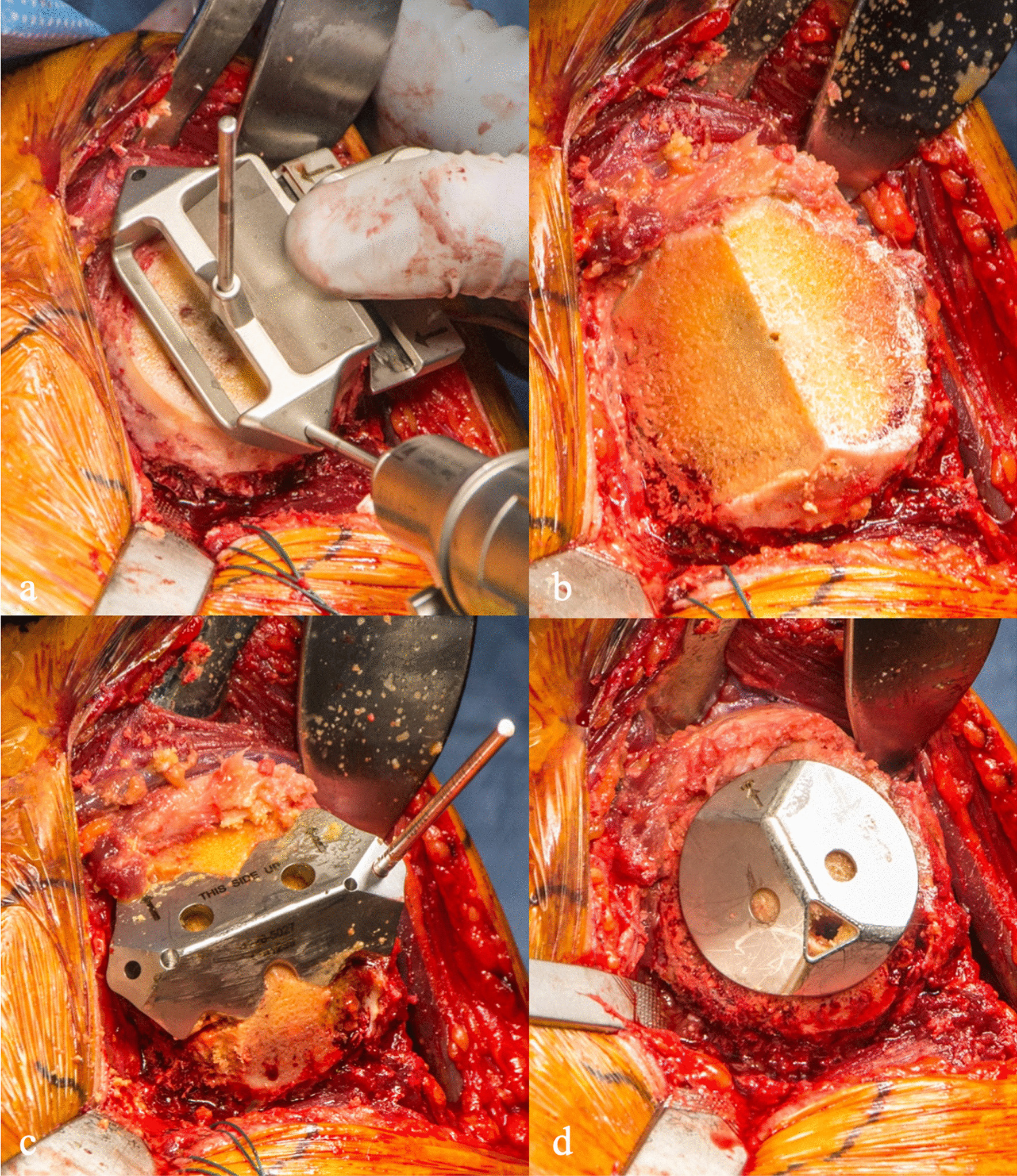
**a** First humeral cut guide is placed, resulting in **b** symmetrical anterior and posterior cut surfaces. **c** second cut guide is then placed allowing the superior and inferior cuts to be made. After completion of humeral preparation, a **d** cut protection plate is placed

A Darrach retractor replaces the Richardson retractor, and is placed medially into the glenohumeral joint and retracts the pectorals major. A cobra retractor replaces the deltoid retractor, and is placed deep to the deltoid but above the subscapularis and supraspinatus (Fig. 2a). A Chandler retractor is then placed under the superior subscapularis. Pressure is applied simultaneously to all 3 retractors as the arm is externally rotated and abducted, resulting in exposure and subsequent dislocation of the humeral head (Fig. 2b). During this process, the Chandler retractor “flips” the subscapularis tendon over the superior aspect of the humeral head. Continued external rotation results in further exposure of the humeral head, which also facilitates removal of inferior and posterior humeral head osteophytes with a rongeur (Fig. 2c). A starting 3.2-mm guidewire pin is drilled under power into the center of the articular surface. The pin guide is then removed, leaving the pin in the center of the humerus. A canulated plunge reamer is then placed over the guidewire pin and roughly 5 to 8 mm of subchondral bone is removed, depending on the radius of curvature of the humeral head (Fig. 2d). The first cut guide for the anterior and posterior cuts is placed using four short pins (Fig. 3a**)**. The guide pin can then be removed to prepare for cuts. The cuts should make a symmetrical edge (Fig. 3b). The second cut guide is then placed and held in position with one to two short pins. The superior and inferior cuts can then be made (Fig. 3c). A 6-mm drill is then used to make four holes for the implant pegs. The pins and the top portion of the drill guide are then removed, and a rongeur is then used to round the four corners at the periphery. Humeral implants are then trialed for size, and once the appropriate size is discovered, the prepared humerus can then be covered with a protection plate designed to match the cut surface (Fig. 3d).

### Glenoid preparation and implant placement

**Fig. 4:**
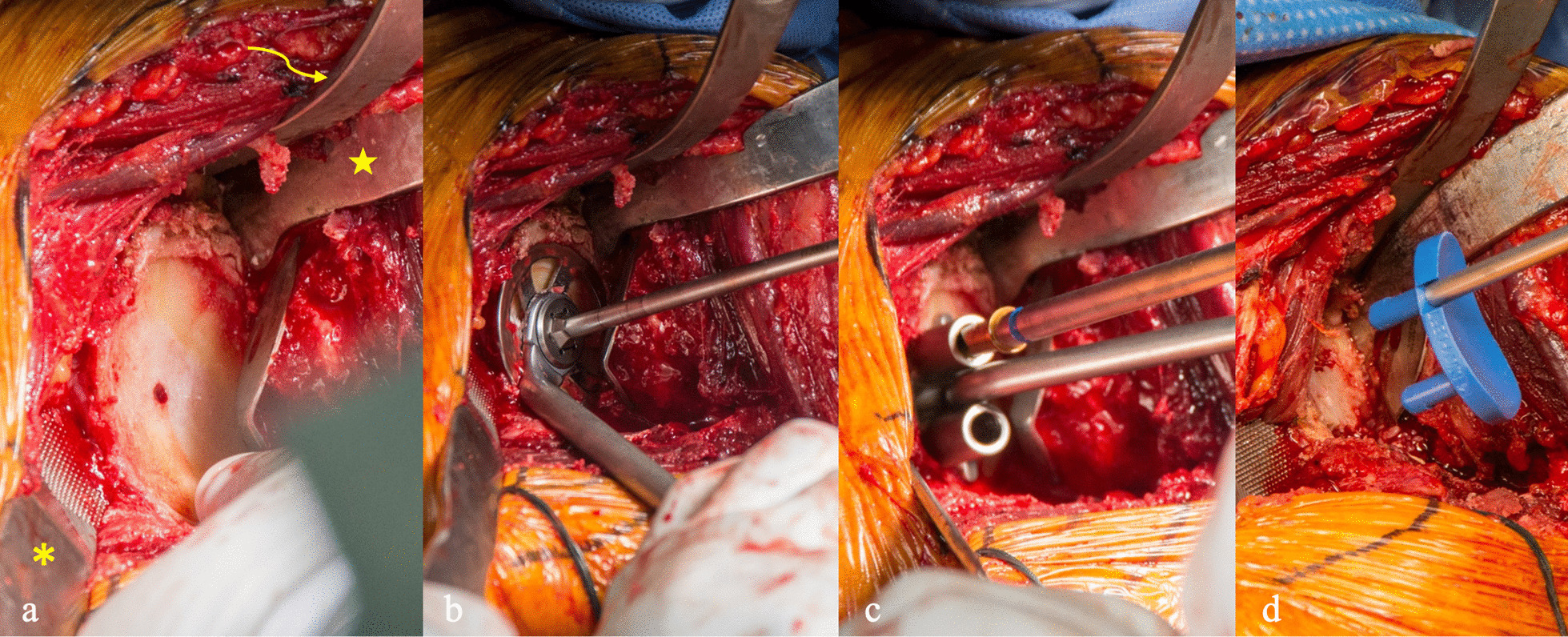
**a** The glenoid is exposed using a Darrach medially (asterisk), small finger superiorly (curved arrow), and a Chandler posterosuperiorly (star), followed by creation of a central pilot hole with the assistance of a glenoid sizing guide. With the use of 17-degree angled tools, **b** reaming, **c** drilling, and **d** trialing of the glenoid is then performed

Glenoid preparation is begun by returning the arm to a neutral rotation position. The glenoid can then be exposed using a Darrach medially, small finger superiorly, and a Chandler posterosuperiorly, and the upper 1/2 of the subscapularis tendon is retracted with an Allis clamp (Fig. 4a). The posterosuperior Chandler retractor provides humeral head distraction for glenoid preparation. A glenohumeral joint capsulectomy is performed from the 12 to 6 o’clock position, and the anterior labrum is removed. This amount of glenoid exposure use is adequate for glenoid preparation with specifically designed angled glenoid instrumentation. At this point, an angled glenoid sizing guide can be used to determine the appropriate glenoid component size, and with the correct sized glenoid guide in place, an awl is used to create a pilot hole in the center of the guide (Fig. 4a). The corresponding sized angled reamer was then used to smooth the glenoid articular surface (Fig. 4b). An angled drill guide can then be placed in the previous guide hole. Two anterior 6-mm holes can be drilled as well as a posterior 4.5-mm hole (Fig. 4c). Trialing of the glenoid can then be done, and an augmented glenoid component can be utilized in the case of a retroverted glenoid (Fig. 4d). At this stage, the arm can be gently internally rotated and extended which can relax the posterior capsule allowing for enough humeral head distraction to bring the glenoid implant in from a slightly anterior position. The glenoid final component is then placed using a third-generation cementing technique, and retractors are then removed.

### Humeral component placement, subscapularis repair, and postoperative protocols

**Fig. 5:**
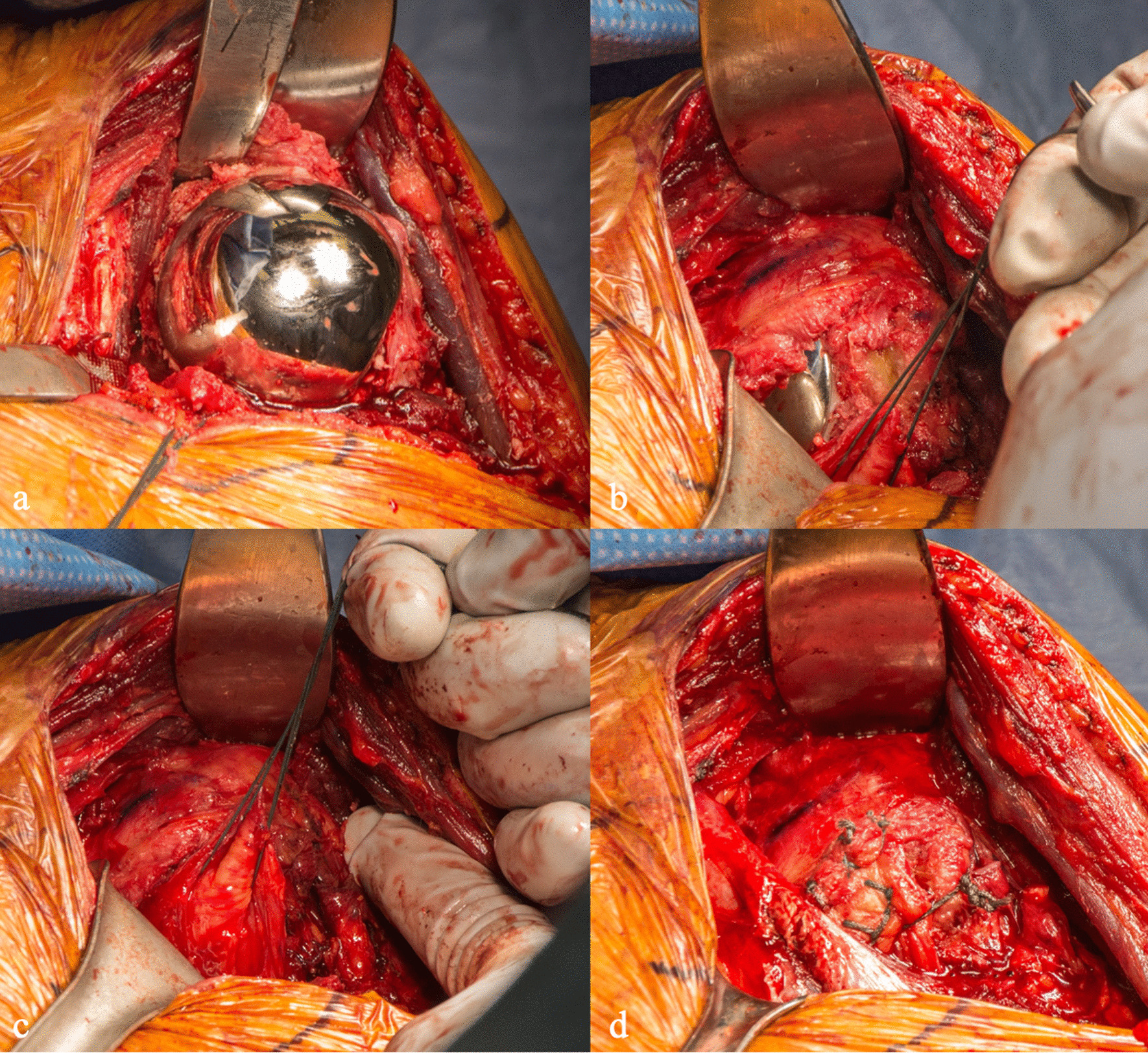
**a** Final humeral head implant is placed, followed **b** reduction into the joint. **c**, **d** The inferior subscapularis flap is reapproximated using multiple No. 5 Ethibond figure-of-8 sutures and a suture anchor seated in the lesser tuberosity

**Fig. 6:**
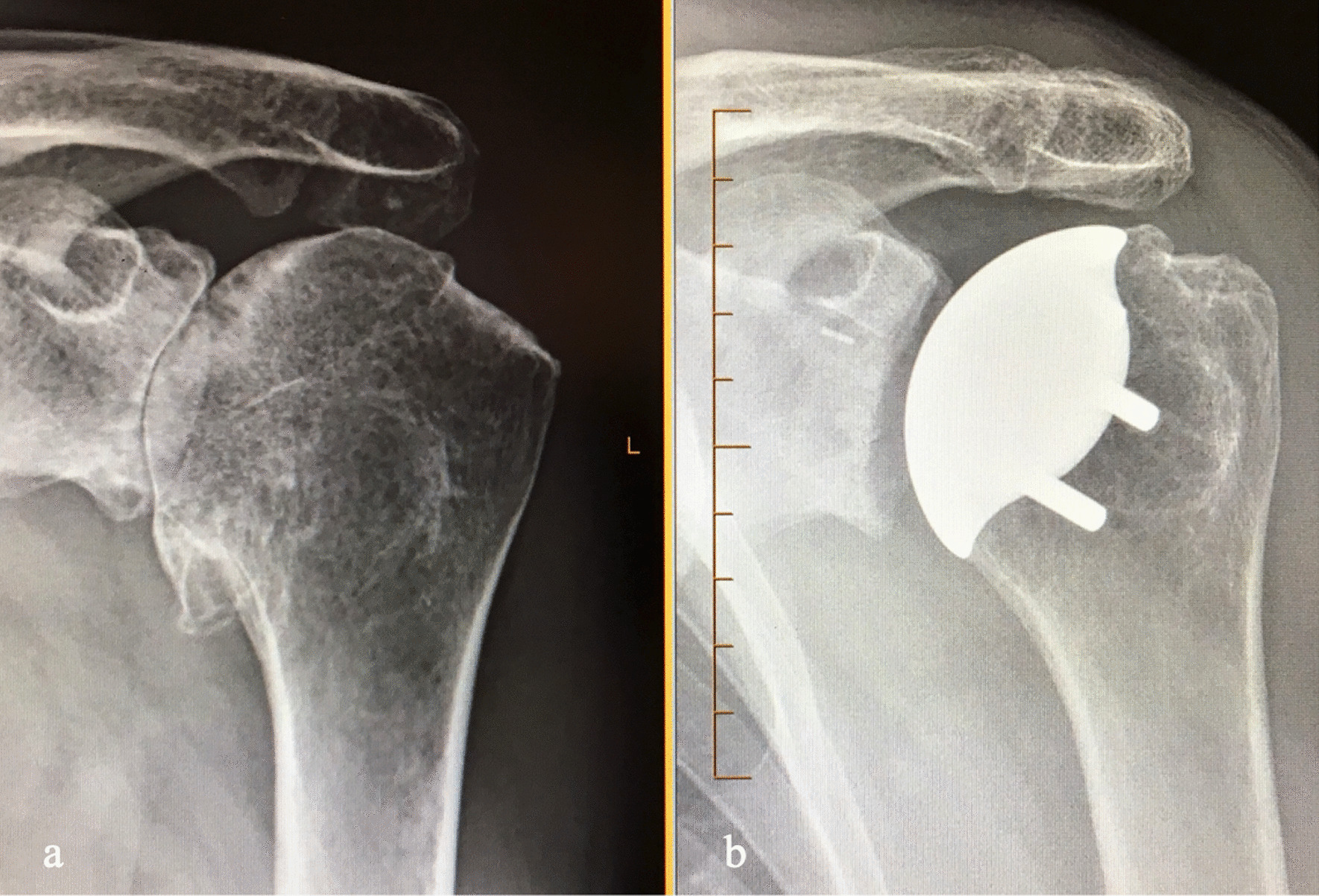
**a** Pre- and **b** Postoperative true AP radiographs with no evidence of missed inferior humeral neck osteophytes

Retractors previously used for humeral head exposure can be used, and humeral head trialing is performed. Once appropriate sizing has been achieved, the humeral head implant is placed using press fit or cemented design (Fig. 5a). The humeral head is reduced into the glenohumeral joint. A cobra retractor can be placed superiorly above the intact subscapularis and a Richardson can be used to retract anteriorly, facilitating repair of the subscapularis (Fig. 5b). The inferior subscapularis flap, previously tagged, is held in place and reapproximated using multiple No. 5 Ethibond figure-of-8 sutures, Mason-Allen-type sutures, and a suture anchor or #5 Ethibond through bone tunnel seated in the inferior lesser tuberosity. (Fig. 5c, d). A drain is not typically placed in the wound, and the patient is placed in a standard sling postoperatively. Postoperative true anteroposterior radiographs are taken in PACU. (Fig. 6a, b).

### Rehabilitation and follow-up

Following the surgery, all patients are scheduled for 1-week, 6-week, 12-week, 6-month, 1-year, and 2-year evaluations. Patients follow an accelerated rehabilitation protocol as a result of decreased intraoperative subscapularis disruption. Patients are initially placed in a sling but are instructed to remove as tolerated, as early as the first postoperative week. Physical therapy is started at week 1 with a four-phase progression. Passive range of motion (ROM) and active external rotation ROM is started at 1 week, and active internal rotation ROM is started at 3 weeks. Isometric rotator cuff strengthening is started around 4 to 6 weeks. New radiographs are taken at the 6-week, 1-year, and 2-year follow-up.

## Discussion

In this technique, a stemless anatomic TSA is placed with the use of angled glenoid instruments through a subscapularis-sparing deltopectoral approach. This technique is presented to demonstrate the possibility for surgeons to capitalize on the improved patient outcomes that are afforded by subscapularis-sparing approaches, while still utilizing the deltopectoral interval to perform a total glenohumeral joint arthroplasty.

A subscapularis takedown has historically been performed in patients undergoing TSA using either a tenotomy, tendon peel, or lesser tuberosity osteotomy. The traditional subscapularis takedown techniques have been compared extensively, with no definitive evidence demonstrating superiority of any of the three approaches [16–18]. The incidence of subscapularis failure with any of these standard approaches, however, has been well-documented [2–4, 6, 19]. Subscapularis-sparing techniques have been employed in an attempt to improve outcomes in total shoulder arthroplasty and have significant potential to reduce the length of rehabilitation, return to work timeline, and total cost to the healthcare system. Three different subscapularis-sparing techniques were identified by Lafosse et al., Simovitch et al., and Savoie et al., respectively, all aiming to preserve the subscapularis tendon attachment and decrease the incidence of subscapularis failure.

The approach described by Savoie et al., which incises the inferior 30–50% of the subscapularis tendon humeral insertion, takes advantage of the fact that the superior subscapularis tendon insertion has a wider anatomic footprint and is thus believed to provide the majority of the subscapularis tendon’s tensile strength [10, 20]. A biomechanical study supported this idea, showing that preservation of the superior 50% of the subscapularis demonstrates a more than doubled load to failure compared to the standard complete tendon peel and repair. This understanding allows surgeons to maintain subscapularis-sparing benefits, while avoiding the pitfalls associated with performing the arthroplasty through the rotator interval. Surgeons are able to use the deltopectoral interval, which allows complete visualization of the humeral head for accurate placement of the humeral component, and also allows complete resection of inferior humeral head osteophytes. The primary pitfall of the approach described by Savoie et al., however, was the inability to adequately achieve glenoid exposure and perform a total joint arthroplasty. This was addressed in the present technique by the use of a unique TSA system with specially designed instruments.

The TSA system utilized in this technique has been previously shown in both clinical and cadaveric studies to result in accurate humeral head placement and sizing through the use of precision multiplanar humeral osteotomy technology [21, 22]. More importantly, however, is the utilization of angled glenoid instruments in this system. The glenoid component sizing guide and drill guide are angled 17 degrees anterior to the glenoid surface, while traditional instruments are straight and must be held directly perpendicular to the articular surface. The angled instruments can instead be held in line with the deltopectoral incision, and they facilitate glenoid preparation without the need for a humeral anatomic neck osteotomy, excessive external rotation, or large soft tissue releases. The glenoid reamer uses a ball-hex drive shaft that allows for polyaxial reaming from an angle requiring less posterior retraction of the humerus, and the angled peg design allows placement without extensive force on a posterior glenoid retractor. Likely, humeral preparation and component placement can be performed through the Savoie subscapularis-sparing surgical approach with essentially any TSA system, but glenoid preparation and component placement may only be feasible at this time using angled glenoid instruments such as those described in this technique.

The use of the Savoie subscapularis-sparing approach introduces the possibility for accelerated rehabilitation protocols. The initial series presented by Savoie et al. allowed passive ROM and active external rotation at 1 week, and active internal rotation ROM starting at 6 weeks [10]. Physical therapy and strengthening as tolerated were begun at 4 weeks, with many patients resuming normal gym activity by 8 weeks, and ultimately all 19 subscapularis tendons were intact at most recent follow-up between 2 and 5 years postoperatively [10]. This same protocol is followed in the technique presented herein, due to the evidence available of being able to safely return patients to their normal daily activity on an accelerated timeline with fewer postoperative restrictions.

## Conclusion

This technique using a TSA system with a polyaxial glenoid reamer and angled pegs on the backside of the glenoid allows the potential for maintenance of the strong postoperative radiographic and patient-reported outcomes that are achieved using traditional TSA approaches, with the advantage of accelerated rehabilitation protocols and decreased risk of subscapularis insufficiency that result from the use of subscapularis-sparing approaches.

## Data Availability

Not applicable.
